# Supplementary figures and images for: Evaluating the transmission dynamics and host competency of aoudad (Ammotragus lervia) experimentally infected with Mycoplasma ovipneumoniae and leukotoxigenic Pasteurellaceae
Source: PLoS One. 2024 Jul 1;19(7):e0294853. doi: 10.1371/journal.pone.0294853 (PMC11216757; doi:10.1371/journal.pone.0294853)

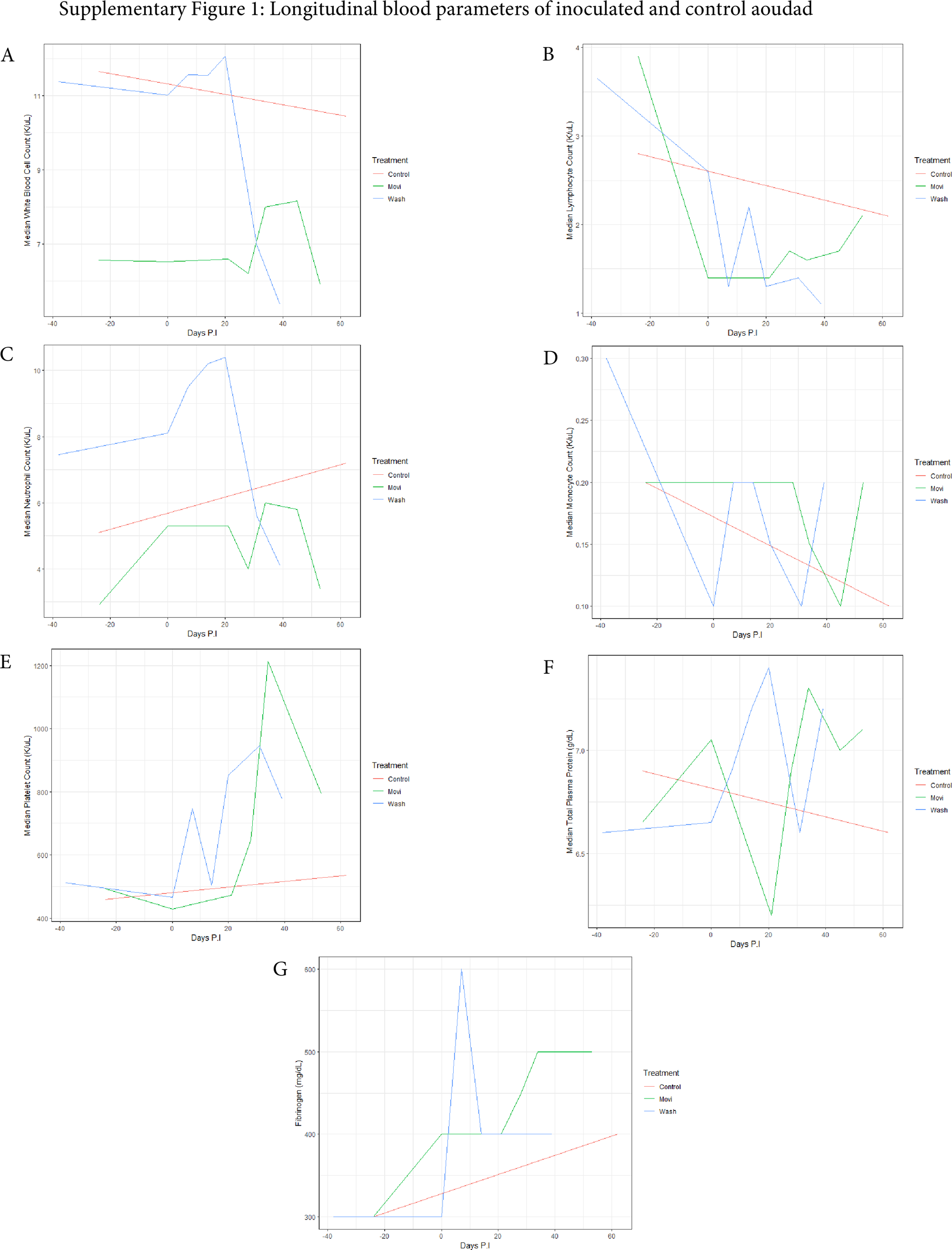

Supplement: S1 Fig — Longitudinal assessment of: A, median white blood cells (K/uL); B, lymphocytes (K/uL); C, neutrophils (K/uL); D, monocytes (K/uL); E, platelets (K/uL); F, total plasma proteins (g/dL); and G, fibrinogen (mg/dL). The group level median of each value was assessed for each day P.I (days post-inoculation. Movi group aoudad were inoculated with only Mycoplasma ovipneumoniae; Wash group aoudad were inoculated with Mycoplasma ovipneumoniae-containing domestic sheep nasal washes; control-contact aoudad were not inoculated, but were allowed contact with the other groups. (TIF) [file pone.0294853.s001.tif]
