# Supplementary material for: Evaluating the transmission dynamics and host competency of aoudad (Ammotragus lervia) experimentally infected with Mycoplasma ovipneumoniae and leukotoxigenic Pasteurellaceae
Source: PLoS One. 2024 Jul 1;19(7):e0294853. doi: 10.1371/journal.pone.0294853 (PMC11216757; doi:10.1371/journal.pone.0294853)
Supplement: S1 Appendix — Multi-locus sequencing results for the inoculum and aouad swabs. This includes descriptions of failed amplification and non-converged sequences. (DOCX) [file pone.0294853.s002.docx]

**Multi-Locus Sanger Sequencing Appendix**

Inoculum sequencing

- **Sample C32** is a nasal swab from a rocky mountain bighorn sheep (*Ovis canadensis canadensis*) that tested positive upon culture and qPCR at Wyoming State Veterinary Laboratory. This aliquot tested at a low-level positive (35. 41) and indeterminate (36.71) cycle threshold value.
  - When sent to the Washington Animal Disease Diagnostic Laboratory, sample C32 was not detected by qPCR and no amplification was observed upon multi-locus Sanger sequencing.
- **Domestic sheep nasal swab samples** were collected to confirm *Mycoplasma ovipneumoniae* colonization. The following are the qPCR results from the Washington Animal Disease Diagnostic Laboratory:
  - 19035 Not detected
  - 19047 Detected Cycle threshold value: 34.6268
  - 19368 Not detected
  - 19372 Not detected
  - 19377 Detected Cycle threshold value: 29.2789
  - 19379 Detected Cycle threshold value: 29.3366
  - 19385 Indeterminate Cycle threshold value: 37.0615
  - 19391 Detected Cycle threshold value: 31.7599
  - 19393 Detected Cycle threshold value: 29.1695
  - 19396 Detected Cycle threshold value: 34. 6345
  - Domestic sheep 19391, 19393, 19377, and 19379 were washed at the nares for the Wash group aoudad inoculum source
- The **nasal washings from domestic sheep 19391, 19393, 19377, and 19379** were then subjected to multi-locus Sanger sequencing at the Washington Animal Disease Diagnostic Laboratory. However, only washings from domestic sheep 19391, 19393, and 19377 amplified. Below are the obtained sequences:
  - **19391**
    - IGS: No amplification
    - LM: No amplification
    - rpoB:
      - gctaaccgtgcacttatgggttcaaacatgcaacgtcaagccgttccgttaattaaaccagaggcaccacttgttgcaacaggaattgaagcagatattgcccgtttttcagcaacaaatattcgtgcccttgaagatggaaaagtcatttttgttgatgcaaaaaaaatcgttgttaattccgataattcaacaaaaacctattttttacgggcttttgaaaaatcaaaccaagaaacattaattttacaaaaaccaactgtaaaagtcggtgacttggttaaaaaaggtcaactaatttgcgatggtccatcaaccgaaaatggtgaactcgcccttggtaaaaatgtgcttgttgcttttagtacttgatatggctacaactacgaagatgccatcataatcagcgaaaagctcgtaaaagacgacgtttttacttcaattcacattcaagaacagacaataaaattccgttcatctaaggcaggaaatgacattctaaccgctgaagtgccaaatgcttcggccagatcacgtgctcatcttgatgcaaatgggattg
    - gyrB:
      - aagcactgttcttgaacggtttttagcggaaaatccagaaaatgccgataaaattattaaaagaaatctgctagctcaaaaagcagctcgggcagctaaatctgcacgcgaagcagtaaagcgtaaatcagcctttgatgtaggaacgcttccaggaaaacttgctgattgttcaacaaaagatccccaaatcgccgaattatatatagttgaggggaactcagctggtgggagtgccaaaatgggtcgtgatcgtaattttcaagcaattttgcctttgcgaggaaaagtgattaactcgcaacgctttcaacttgaaaaagttttacgcaatgaagaaattatttcaatgatcaccgcttttggtactggagttggacctgaatttaatattagtaaa
  - **19393**
    - IGS: No amplification
    - LM: No amplification
    - rpoB: No amplification
    - gyrB:
      - aagcactgttcttgaacggtttttagcggaaaatccagaaaatgccgataaaattattaaaagaaatctgctagctcaaaaagcagctcgggcagctaaatctgcacgcgaagcagtaaagcgtaaatcagcctttgatgtaggaacgcttccaggaaaacttgctgattgttcaacaaaagatccccaaatcgccgaattatatatagttgaggggaactcagctggtgggagtgccaaaatgggtcgtgatcgtaattttcaagcaattttgcctttgcgaggaaaagtgattaactcgcaacgctttcaacttgaaaaagttttacgcaatgaagaaattatttcaatgatcaccgcttttggtactggagttggacctgaatttaatattagtaaa
  - **19377**
    - IGS:
      - aattactactaaatgtggttaaaatcaaactaacacattagaaaatttaataatttgaaataatttgttctattaattcattttctccttgtttagttttgtaaatattattttttgtaaagtaaatttttttttaactttttttaatgaattttttaatacaatagcaatttgaaattgattatgtacctagttttgagagctctaaaactctcaaaacgtctgtaaaaagacaattagctctttcaaaactgaacagtaacaattttatatctgatctaaaaatcagataatattccaaagtcttttaaaattaaaccgagtttatttttaaaattttataacttttagttataaaaactctaaaattgttaaaataccttaagatatattatctaaatag
    - LM:
      - tatgttagcttgctaatatatttagtagcaaatgggtgagtaacacgtacctaacctaccttttggaccgggataaccattggaaacagcggctaataccagatatgataaaaaaatgcatgtttttttattaaaagaagcctttaaagcttcaccaaaaaatgggggtgcgcaacattagttagttggtagggtaaaggcctaccaagacgatgatgtttagcggggccaagaggctgtaccgccacactgggattgagatacggcccagactcctacgggaggcagcagtaaggaatattccacaatgagcgaaagcttgatggagcgacac agagtgcag
    - rpoB:
      - gctaaccgtgcacttatgggttcaaacatgcaacgtcaagccgttccgttaattaaaccagaggcaccacttgttgcaacaggaattgaagcagatattgcccgtttttcagcaacaaatattcgtgcccttgaagatggaaaagtcatttttgttgatgcaaaaaaaatcgttgttaattccgataattcaacaaaaacctattttttacgggcttttgaaaaatcaaaccaagaaacattaattttacaaaaaccaactgtaaaagtcggtgacttggttaaaaaaggtcaactaatttgcgatggtccatcaaccgaaaatggtgaactcgcccttggtaaaaatgtgcttgttgcttttagtacttgatatggctacaactacgaagatgccatcataatcagcgaaaagctcgtaaaagacgacgtttttacttcaattcacattcaagaacagacaataaaattccgttcatctaaggcaggaaatgacattctaaccgctgaagtgccaaatgcttcggccagatcacgtgctcatcttgatgcaaatgggattg
    - gyrB:
      - aagcactgttcttgaacggtttttagcggaaaatccagaaaatgccgataaaattattaaaagaaatctgctagctcaaaaagcagctcgggcagctaaatctgcacgcgaagcagtaaagcgtaaatcagcctttgatgtaggaacgcttccaggaaaacttgctgattgttcaacaaaagatccccaaatcgccgaattatatatagttgaggggaactcagctggtgggagtgccaaaatgggtcgtgatcgtaattttcaagcaattttgcctttgcgaggaaaagtgattaactcgcaacgctttcaacttgaaaaagttttacgcaatgaagaaattatttcaatgatcaccgcttttggtactggagttggacctgaatttaatattagtaaa
  - **19379**
    - IGS: No amplification
    - LM: No amplification
    - rpoB: No amplification
    - gyrB: No amplification
- Using The National Center for Biotechnology Information’s blastn-2 sequence tool (https://blast.ncbi.nlm.nih.gov/Blast.cgi?PROGRAM=blastn&PAGE_TYPE=BlastSearch&LINK_LOC=blasthome), we found the following patterns from the available multi-locus sequencing data for sample that amplified:
  - the gyrB locus aligned with 100% identity for samples 19391, 19393, and 19377
  - the rpoB locus aligned with 100% identity for samples 19391 and 19377

Nasal swab sequencing

- Movi group AD #2 nasal swab failed to amplify
- Wash group AD #9 nasal swab failed to amplify
- Wash group AD #13 amplified, but Washington Animal Disease Diagnostic Laboratory was unable to obtain clean sequences
