# Supplementary material for: Evaluating the transmission dynamics and host competency of aoudad (Ammotragus lervia) experimentally infected with Mycoplasma ovipneumoniae and leukotoxigenic Pasteurellaceae
Source: PLoS One. 2024 Jul 1;19(7):e0294853. doi: 10.1371/journal.pone.0294853 (PMC11216757; doi:10.1371/journal.pone.0294853)
Supplement: S2 Appendix — Supplementary details on gross pulmonary and non-pulmonary lesions of inoculated and control aoudad. (DOCX) [file pone.0294853.s003.docx]

**S2 Appendix: Pathology Appendix**

At necropsy, pulmonary findings ranged from acute to chronic and included fibrinous pleuritis, pleural effusion, fibrous pleural adhesions, mucopurulent exudate in the trachea and mainstem bronchi, consolidation of the cranioventral lung lobes, and pulmonary abscessation. Lesions of Movi group AD lesions (5/6 AD) consisted of fibrous pleural adhesions (50%, AD #4, 5, 7) (Fig 2A), cranioventral lung consolidation (33%, AD #1, 6) (Fig 2B), and mucopurulent exudate in the trachea and mainstem bronchi (17%, AD #1) (Fig 2C). One Movi group AD (17%, AD #3) had no gross lesions of pneumonia.

Non-pulmonary gross lesions observed in all groups included serous atrophy of fat (Movi group 33%, Wash group 80%, Control-contact 33%) and caseous lymphadenitis (Movi group 50%, Wash group 20%, Control-contact 100%). Caseous lymphadenitis was observed in the retropharyngeal, superficial cervical, sternal, tracheobronchial, and mesenteric lymph nodes. One AD in the Movi group (17%, AD #7) had pyogranulomatous lymphadenitis of the tracheobronchial lymph nodes caused by *Coccidioides immitis*. Two AD in the Movi group (33%, AD #1, 4) and one of the Control-contact AD (33%, AD #16) had suppurative lymphadenitis affecting the tracheobronchial and mesenteric lymph nodes. Other lesions within the Movi group included *Haemonchus* sp*.* in the abomasum (17% AD #6), *Oesophagostomum* sp. granulomas throughout the intestine (67%, AD #3, 4, 5, 7), and mesenteric cysticercosis caused by *Taenia hydatigena* (33%, AD #3, 7). Mesenteric cysticercosis was also observed in the Wash group (40%, AD #8 and 13). One AD in the Wash group (20%, AD #9) had renal medullary crest necrosis.
