# Supplementary material for: Evaluating the transmission dynamics and host competency of aoudad (Ammotragus lervia) experimentally infected with Mycoplasma ovipneumoniae and leukotoxigenic Pasteurellaceae
Source: PLoS One. 2024 Jul 1;19(7):e0294853. doi: 10.1371/journal.pone.0294853 (PMC11216757; doi:10.1371/journal.pone.0294853)
Supplement: S1 Table — Supplementary details on the pattern and distribution of lesions and pathogen detections for individual aoudad. (DOCX) [file pone.0294853.s004.docx]

| **Aoudad ID** | **Group** | ***M. ovi* lesions** | ***M. ovi* PCR result** | **Bronchopneumonia** | **Oat cells** | **LKT PCR** | **Lung cultures** |
| --- | --- | --- | --- | --- | --- | --- | --- |
| 1 | Movi | + | - | + | - | NA | *Pasteurella multocida, Streptococcus lutetiensis* |
| 3 | Movi | + | - | - | - | - | Not done |
| 4 | Movi | + | + | - | - | NA | *Biberstinia trehalosi, Streptococcus lutetiensis* |
| 5 | Movi | + | - | - | - | - | *Corynebacterium pseudotuberculosis* |
| 6 | Movi | + | NA | - | - | NA | *Acinebacter sp.* |
| 7 | Movi | + | - | - | - | - | *Trueperella pyogenes, Biberstinia trehalos* |
| 8 | Wash | + | + | + | - | + | *Mannheimia haemolytica, Moraxella bovoculi* |
| 9 | Wash | - | - | + | + | + | *Mannheimia haemolytica, Escherichia coli, Acinetobacter sp.* |
| 12 | Wash | - | - | - | - | NA | *M.haemolytica* |
| 13 | Wash | + | - | + | - | - | *Pasteurella multocida, Mycoplasma ovipneumoniae suspect* |
| 14 | Wash | + | + | + | + | NA | *Mannheimia haemolytica, Trueperella pyogenes* |
| 15 | Control | + | - | - | - | - | *Biberstinia trehalosi* |
| 16 | Control | + | - | - | - | - | None isolated |
| 21 | Control | + | - | - | - | - | *Pasteurella multocida* |

S1 Table: **Individual lesion patterns**

Animal ID, identification number of each individual; Group, treatment group of each individual; *M. ovi* lesions, presence or absence of lesions consistent with *Mycoplasma ovipneumoniae* infection; *M. ovi* PCR result, positive versus indeterminate or negative results from *Mycoplasma ovipneumoniae*-specific real-time polymerase chain reaction; Bronchopneumonia, presence or absence of lesions consistent with bronchopneumonia; Oat cells, presence or absence of oat cells; LKT PCR positive or negative results for leukotoxin-A specific polymerase chain reaction; Lung cultures, species isolated from tissues cultured under aerobic and anaerobic conditions; +, present or positive result; -, absent or negative result
